# Genetic analysis of the two zebrafish patched homologues identifies novel roles for the hedgehog signaling pathway

**DOI:** 10.1186/1471-213X-8-15

**Published:** 2008-02-19

**Authors:** Marco J Koudijs, Marjo J den Broeder, Evelyn Groot, Fredericus JM van Eeden

**Affiliations:** 1 Hubrecht Institute, Developmental Biology and Stem Cell Research, Uppsalalaan 8, 3584CT, Utrecht, The Netherlands; 2 University of Sheffield, Department of Biomedical Science, Western Bank, Sheffield S10 2TN, UK

## Abstract

**Background:**

Aberrant activation of the Hedgehog (Hh) signaling pathway in different organisms has shown the importance of this family of morphogens during development. Genetic screens in zebrafish have assigned specific roles for Hh in proliferation, differentiation and patterning, but mainly as a result of a loss of its activity. We attempted to fully activate the Hh pathway by removing both receptors for the Hh proteins, called Patched1 and 2, which are functioning as negative regulators in this pathway.

**Results:**

Here we describe a splice-donor mutation in Ptc1, called *ptc1*^*hu1602*^, which in a homozygous state results in a subtle eye and somite phenotype. Since we recently positionally cloned a *ptc2 *mutant, a *ptc1;ptc2 *double mutant was generated, showing severely increased levels of *ptc1*, *gli1 *and *nkx2.2a*, confirming an aberrant activation of Hh signaling. As a consequence, a number of phenotypes were observed that have not been reported previously using *Shh *mRNA overexpression. Somites of *ptc1;ptc2 *double mutants do not express anteroposterior polarity markers, however initial segmentation of the somites itself is not affected. This is the first evidence that segmentation and anterior/posterior (A/P) patterning of the somites are genetically uncoupled processes. Furthermore, a novel negative function of Hh signaling is observed in the induction of the fin field, acting well before any of the previously reported function of Shh in fin formation and in a way that is different from the proposed early role of Gli3 in limb/fin bud patterning.

**Conclusion:**

The generation and characterization of the *ptc1;ptc2 *double mutant assigned novel and unexpected functions to the Hh signaling pathway. Additionally, these mutants will provide a useful system to further investigate the consequences of constitutively activated Hh signaling during vertebrate development.

## Background

The Hedgehog (Hh) signaling pathway has been the focus of much research in the last two decades, highlighting the importance of this morphogen in the control of patterning, differentiation and proliferation during development and disease [1-3]. The consequences of aberrantly activated Hh signaling have been identified using forward and reverse genetic approaches in vertebrates and invertebrates. Large-scale genetic screens using zebrafish identified mutants encoding several components of the Hh signaling pathway, like Shh [4], Smo [5], Gli1 [6], Gli2 [7], Dispatched [8], dzip1 [9,10] and Scube2 [11,12]. Mutants of the Hh pathway were mainly identified morphologically by their effect on somite development, as they resulted in U-shaped somites. This defect probably results from the absence of adaxial cells or muscle pioneers in the mutant myotome [13]. The proteins encoded by these mutants are mainly positive regulators, where inactivating mutations result in an inhibition of the pathway. Recently, we showed that a class of mutants consisting of *dre*, *uki *and *lep*, encode the negative regulators of Hh signaling, Sufu, Hip, and Ptc2, respectively [14]. These mutants show an increased level of proliferation in different tissues but do not show the usual patterning defects described for Hh overexpression experiments. The slight activation of Hh in these mutants did not result in similar defects as seen by ectopic expression of *shh *[15] or *dnPKA *[16], morphologically resulting in flattened somites. Additionally, optic cup vs. stalk differentiation defects [17], dorsoventral patterning of the brain [18] and neural tube (for a review: [19]), are described to be controlled by Hh signaling. None of these phenotypes could be observed in the *dre*, *uki *or *lep *mutant. Triple mutants for Sufu, Hip and Ptc2 did not show an enhanced phenotype, suggesting that additional regulators are still functioning to inhibit the Hedgehog pathway, most likely Ptc1 [14].

A well-described role for Hh signaling during development involves the specification of the myotome in fast and slow muscle type fibers (for a review: [20,21]. Overactivation of the Hh signaling pathway by *shh *overexpression, results in a complete conversion of the myotome into slow muscle cells, at the expense of fast muscle cells [16,22-25]. The exact genetic program controlled by Hh, underlying this specification, is still largely unknown. Positional cloning of the *ubo *mutant, a member of the u-type mutant class, shed some light on this regulation. It was found that this locus encodes Prdm1 (previously described as Blimp-1) [26]. This gene is a downstream target of Hh signaling [27] and can act as a transcriptional repressor [28]. Ectopic *prdm1 *expression is able to rescue slow muscle development in *smu *mutants, completely lacking Hh signaling [26], indicating a pivotal role for Prdm1 in specifying slow muscle cell identity.

Besides its role in specification and differentiation of muscle cell identity, a well-established role for Hh involves the regulation of A/P patterning of the outgrowing fin buds. The fin buds arise from specified regions of the lateral plate mesoderm (LPM) by a cascade of different genes. One of the earliest factors involved in fin field induction is Vitamin A derived Retinoic acid (RA), produced by Aldh1a2. Gibert et al, [29], have shown that RA produced in the somitic mesoderm is necessary during early segmentation (6 to 8 somite stage) for proper fin induction. The RA signal is thought to be transferred to the intermediate mesoderm where it activates Wnt2b [30]. Subsequently, the T-box transcription factor *tbx5 *[31], one of the earliest genes known to be expressed in the fin field, is induced in the LPM under control of *wnt2b *[32]. In the fin mesenchyme, *tbx5 *induces, among other genes, *fgf24, fgf10 *and *prdm1 *expression to further specify the fin primordium. *fgf24 *induces the expression of *shh *in the posterior mesenchyme of the fin bud [33] called the zone of polarizing activity [34], which organizes the A/P patterning of the outgrowing fin. The importance of Hh for zebrafish fin development became apparent in the *syu *and *smu *mutant that encode Shh and Smoothened, respectively. These mutants do form fin buds, but subsequently fail to grow out correctly [5,35]. On the other hand, a slight activation of Hh signaling, as described for the *uki*^*hu418b *^mutant, results in enlarged pectoral fins, probably as a result of increased proliferation within the developing fin bud [14]. These data show that Hh is functioning rather late in the genetic program controlling pectoral fin development.

Here we describe the identification of two *ptc1 *alleles, showing subtle Hh overexpression phenotypes, affecting eye and somite development. In contrast, zebrafish mutants for both *ptc *genes show severe developmental problems, indicating redundancy between the two Ptc homologues. We find that constitutive activation of Hh signaling negatively regulates the induction of the pectoral fin field. The Hh signaling pathway therefore acts significantly earlier in fin field induction than its well-described role in A/P patterning of the outgrowing fin bud. Additionally, a negative role for Hh signaling in the specification of A/P patterning of the somites is observed, where somites of the *ptc1;ptc2 *mutants appear to be apolar, without affecting segmentation. This is the first evidence that A/P patterning and segmentation are uncoupled processes. The described mutants assign novel roles to Hh signaling during development and will be of major importance for further studies focusing on the effects of constitutive activation of Hh signaling during development and disease.

## Results

### Identification and recovery of a splice-donor mutation in the *ptc1 *gene

The *dre*, *uki*, and *lep *mutant class has shown that multiple regulators secure the activity of the Hh signaling pathway. This was illustrated by the fact that the concurrent loss of function of the negative regulators Sufu, Hip and Ptc2 did not result in the typical Hh overexpression phenotype, mainly concerning the chevron shape of the somites [14]. Presumably, the slight increase in Hh signal is enough to increase Ptc1 expression via a sensitive auto-regulatory loop, thereby maintaining the pathway at a certain level of activity, which does not exceed the threshold for Hh activation that will lead to patterning defects.

**Figure 1:**
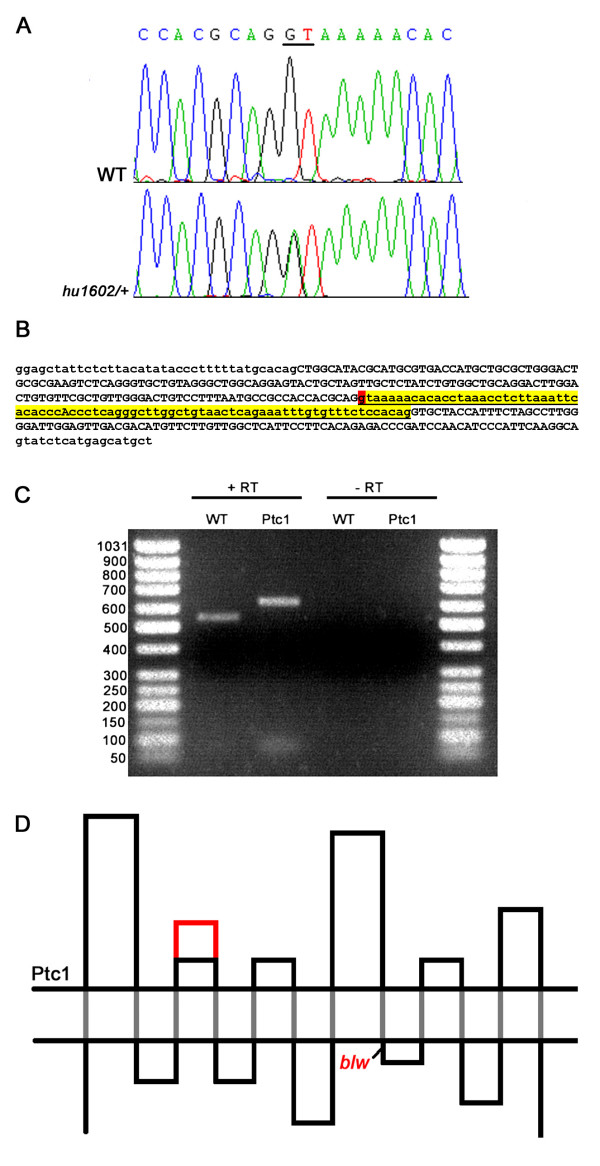
**Identification and characterization of a splice-donor mutation in the zebrafish *ptc1 *gene**. A splice donor mutation was identified in the first base pair of intron 10, changing the consensus sequence GT to AT, probably affecting splicing (A). The intron after exon 10, shown in lower case in yellow, contains 81 bp, and the splice donor position mutated in *ptc1*^*hu1602 *^is indicated in red (B). RT-PCR analysis confirms that splicing is affected as a result of the mutation, which extends the transcript with 81 bp compared to wild type (C). Schematic representation of the Ptc1 protein, showing the 12 transmembrane domains (black dots), and the extension of the second extracellular loop with 27 AA in red. The *blw *mutation is positioned directly after the eighth transmembrane domain of the protein (D).

To test this hypothesis we have generated a target-selected knockout of the Ptc1 gene. An ENU-induced mutation library of approximately 12,000 F1 fish was screened for mutations in this gene using the TILLING method [36]. This resulted in the identification of a splice donor mutation, changing the 5' consensus sequence of the intron after exon 10, from GT, to AT (Fig. 1A, referred to as *ptc1*^*hu1602*^). The removal of this splice site results in the insertion of the 81 bp intron into the transcript (Fig. 1B). RT-PCR (Fig. 1C) and sequencing experiments on a fragment containing exons 10 to 13 confirmed that this complete intron is inserted in frame into the ORF, resulting in an expansion of the second extracellular loop of the Ptc1 protein by 27 amino acids (Fig. 1D).

### *ptc1*^*hu1602 *^mutants show an eye and somite phenotype

**Figure 2:**
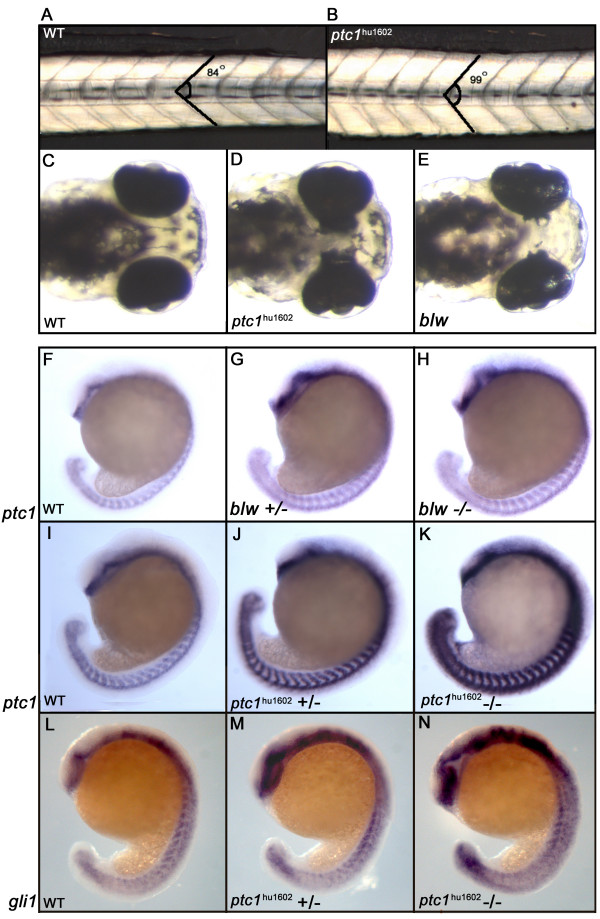
**Phenotypic consequences of zebrafish *ptc1 *mutants**. Homozygous *ptc1*^*hu1602 *^mutants show a subtle somite phenotype at 32 hpf, where the average angle of the somite becomes more obtuse (A,B). At 72 hpf, *ptc1*^*hu1602 *^mutants exhibit an eye phenotype where the pigmented epithelium is extended into the diencephalon. The similar phenotype described for the *blw *mutant is weaker compared to the *ptc1*^*hu1602 *^mutant (C-E). The expression level of *ptc1*, a general readout for Hh activity, shows a mild increase in the *blw *mutant compared to wild type (F-H). The *ptc1*^*hu1602 *^mutant shows a severely increased level of *ptc1*, where wild types, heterozygotes and mutants can be distinguished based on *ptc1 *levels (I-K). Additional to the difference in the strength of the eye phenotype, the activation of the pathway is significantly higher in *ptc1*^*hu1602 *^mutants compared to *blw *mutants. An increased expression level of *gli1 *confirms an activation of the Hh pathway in the *ptc1*^*hu1602 *^mutant (L-N).

Zebrafish homozygous for *ptc1*^*hu1602 *^display a subtle somite phenotype at 32 hours post fertilization (hpf), where the angle of the chevron shaped form of the somites becomes more obtuse (Fig. 2A,B). The average angle of the somite is 84° in a wild type situation (n = 6, 4 somites measured), compared to 99° for the *ptc1*^*hu1602 *^mutant (n = 6, 4 somites measured). However, the typical flattened somite phenotype, as observed after Shh overexpression in zebrafish, was not observed, probably due to redundancy with Ptc2. Additionally, at 72 hpf a partially penetrant eye phenotype is observed, where the retinal pigmented epithelium (RPE) extends into the diencephalon (Fig. 2C,D). This phenotype has already been described for the *blowout *(*blw*^*tc294z*^) mutant [37], suggesting that blowout may also be a mutation in *ptc1*. Complementation analysis indicated that *ptc1*^*hu1602 *^and *blw*^*tc294z *^are allelic (data not shown). Subsequent sequence analysis of all exons of *ptc1 *identified a premature stop codon (W1039X) in the *blw*^*tc294z *^allele, located after the eighth transmembrane domain (Fig. 1D). Comparing *ptc1*^*hu1602 *^with *blw*^*tc294z *^revealed that *ptc1*^*hu1602 *^has a higher penetrance of the eye phenotype (21.4% in total, 3.4% single eye, 18% both eyes affected, n = 261) than *blw*^*tc294z *^(5% in total, 4.4% one eye, 0.6% both eyes, n = 521). Additionally the RPE in the *ptc1*^*hu1602 *^extends more severely into the diencephalon of the embryo compared to *blw*^*tc294z *^(Fig. 2E), suggesting that the *ptc1*^*hu1602 *^allele is stronger. As transcription of *ptc1 *is known to be upregulated by increased Hh signaling, we decided to test whether our mutations resulted in an increase in *ptc1 *transcripts. *In situ *hybridization (ISH) experiments showed that both alleles resulted in an increase in *ptc1 *transcripts. In addition, the *blw*^*tc294z *^mutant (Fig. 2F–H) shows milder upregulation than the *ptc1*^*hu1602 *^mutant (Fig. 2I–K) at 18 hpf, again indicating that the latter allele is stronger. An alternative explanation for this observation might be that the splice donor mutation in *ptc1*^*hu1602 *^increases the stability of the *ptc1 *transcript, subsequently resulting in a higher expression level after ISH. Therefore we analyzed the expression levels of *gli1*, another downstream transcriptional target of Hh signaling [6] to confirm the overactivation of the pathway in the *ptc1*^*hu1602 *^mutant. The expression levels of *gli1 *at 19 hpf distinguishes wild types from heterozygotes and mutants (Fig 2L–N). This confirms that the pathway is indeed activated, and that the increase in *ptc1 *expression is not a consequence of altered stability of the transcript, due to the induced mutation, but a representation of the activity of Hh signaling in this mutant.

The *ptc1*^*hu1602 *^allele results in an in frame insertion of 27 amino acids into the protein sequence, and therefore we investigated whether this is a full null or a partial loss of function allele. To test this hypothesis we injected a MO against *ptc1 *[25] into a *ptc1*^*hu1602 *^mutant background, which did not enhance the phenotype. Additionally, we checked whether the *ptc1*^*hu1602 *^allele has a dominant negative effect, by injecting wild type embryos with *ptc1*^*hu1602 *^mRNA. No phenotypes comparable with the *ptc1*^*hu1602 *^mutant were observed, excluding a dominant negative effect. Together the data suggest that *ptc1*^*hu1602 *^is at least a strong loss of function allele. Since the *ptc1*^*hu1602 *^allele is stronger than *blw*^*tc294z*^, we used this allele for subsequent analysis and therefore we will refer to *ptc1*^*hu1602 *^when *ptc1 *is mentioned.

### The Hh signaling pathway is constitutively activated in *ptc1;ptc2 *mutants

**Figure 3:**
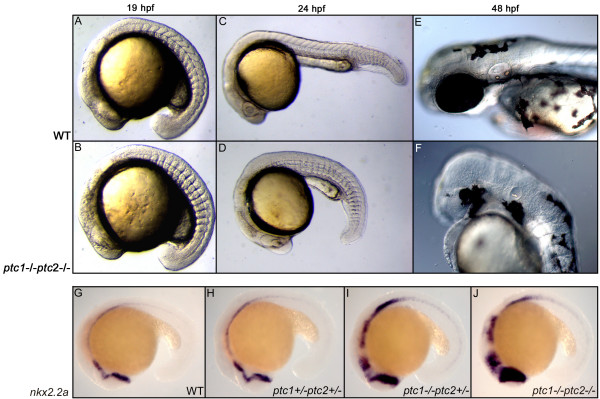
**Concurrent inactivation of *ptc1 *and *ptc2 *results in severe developmental defects**. At 18 hpf, a somite phenotype becomes apparent in the *ptc1;ptc2 *double mutants, where the chevron shaped form of the somites becomes straight (A,B), which is a typical consequence of increased activity of the Hh pathway. At 24 hpf, *ptc1;ptc2 *double mutants do not develop a lens but the primitive eye field is still present (C,D). At 48 hpf the eyes are completely absent. Additionally, reduced pigmentation, an absence of the nose, and an underdeveloped ear can be observed at 48 hpf (E,F). Expression levels of *nkx2.2a *confirms that the pathway becomes more activated upon losing wild type alleles of *ptc1 *or *ptc2*, with the highest expression in the *ptc1;ptc2 *mutant, mainly in the anterior brain structures (G-J).

By the identification of a zebrafish *ptc1 *mutant we are now able to investigate whether the inhibition of the pathway is dependent on the presence of both Ptc homologues. Based on the current model, the absence of both Ptc proteins will constitutively activate Smo, normally inhibited by Ptc, which in turn activates the entire downstream pathway. Double mutants for *lep(ptc2)*^*tj222 *^and *ptc1*^*hu1602 *^were generated (hereafter referred to as *ptc1;ptc2 *mutants) and these were expected to show the consequences of increased Hh signaling. From previous studies it has been shown that overactivation of the Hh pathway, after injection of Shh or dnPKA [16,25], results in a flattened somite phenotype. Phenotypically, *ptc1;ptc2 *mutants clearly exhibit a flattened somite phenotype at 18 hpf (Fig. 3A,B) confirming an activation of the pathway, by losing both Ptc homologues. At 24 hpf, the primitive eye field is present but the lens is missing (Fig. 3C,D), where at 48 hpf a complete absence of the eyes and the nose could be observed (Fig. 3E,F). To confirm an activation of the pathway we analyzed the expression level of *nkx2.2a *[15] and *gli1 *[6]. The expression of *nkx2.2a *is only induced when Hh activity is high. A clear increase in *nkx2.2a *(Fig. 3G–J) *ptc1 *and *gli1 *(See Additional file 1) expression could be observed between the different genotypes, confirming that the activity of the Hh signaling pathway is severely upregulated in *ptc1;ptc2 *mutants. Administration of 10 mM of cyclopamine to *ptc1;ptc2 *partially rescued the phenotype and resulted in a decrease in *ptc1 *expression levels (data not shown). This confirms that the phenotype seen in *ptc1;ptc2 *mutants is a result of increased Hh pathway activation.

The eye phenotype of a single *ptc1*^*hu1602 *^mutant, a protrusion of the pigmented epithelium towards the medial region of the brain, could be a result of a disturbed balance between optic cup versus optic stalk differentiation. This process is reported to be under control of Hh signaling [17,38]. Possibly, the absence of the eyes in the *ptc1;ptc2 *double mutants could be a further disturbance of cup versus stalk differentiation. To test this hypothesis, we analyzed the expression pattern of *pax2 *and *pax6*, identifying the presumptive optic stalk and cup respectively. Indeed, *ptc1;ptc2 *mutants expand *pax2 *expression and lose *pax6 *expression in the presumptive optic cup region (See Additional file 1), confirming a differentiation defect during eye development. Finally, *ptc1;ptc2 *mutants show an expansion of the lateral floor plate, indicated by *foxa *ISH, whereas *shh *expression in the medial floor plate was unaffected (See Additional file 1). Again, this is in line with previous reports [39], and confirms the overactivation of the Hh pathway in the *ptc1;ptc2 *mutants. These observations justify the assumption that the Hh pathway can be constitutively activated in a genetic manner, by losing activity of both Ptc homologues.

### *ptc1;ptc2 *mutants show mediolateral and anteroposterior patterning defects

**Figure 4:**
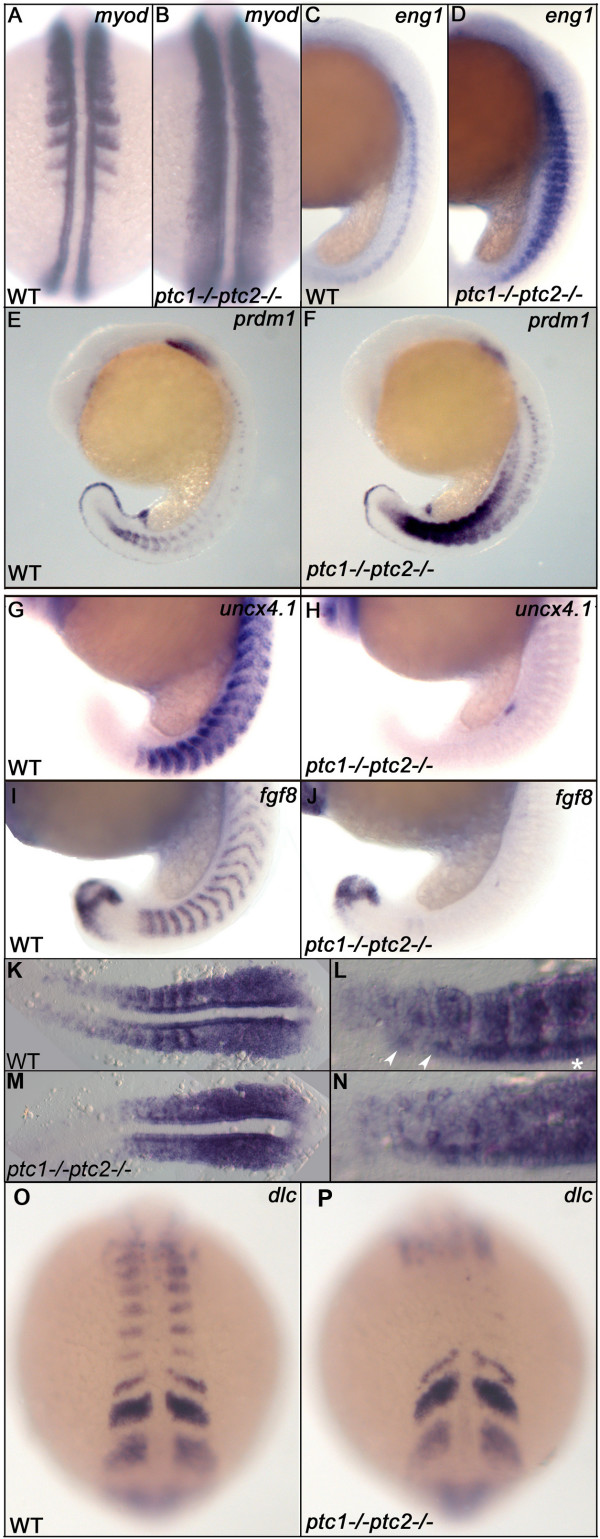
***Ptc1;ptc2 *double mutants show mediolateral and anteroposterior somite patterning defects**. Patterning of adaxial cells and slow muscle cell precursors is disturbed in *ptc1;ptc2 *mutants. The region of *myod *positive adaxial cells and *eng1 *expressing slow muscle precursors are expanded at 19 hpf (A-D). At 19 hpf, *prdm1 *expression is highly induced in the *ptc1;ptc2 *mutant, suggesting that the myotome is mainly developing slow muscle type precursors (E,F). Anteroposterior patterning of the somites is lost in segmented somites, since the posterior somite marker *uncx4.1 *(G,H) and anterior somite marker *fgf8 *(I,J) are strongly reduced or not detectable at 19 hpf. *myf5 *expression in 11 somite stage wildtype (K,L) and *ptc1;ptc2 *double mutant embryos (M,N). L and N are higher magnification of relevant areas of K and M, respectively. In wild type, *myf5 *is expressed at higher levels in the posterior of the somites during their formation, in more posterior (younger) segments this appears to include the adaxial cells (*). In more anterior (more mature) somites more anterior adaxial cells appear to show higher levels of labeling (arrowheads). In *ptc1;ptc2 *double mutant embryos (N) a "salt and pepper" type staining suggests that anterior and posterior cells are intermingled. Additionally, *dlc *necessary for proper segmentation is present in presomitic mesoderm but failed to be expressed in the posterior part of segmented somites (O,P), suggesting that somite formation and A/P patterning of formed somites are genetically uncoupled processes.

In zebrafish, somites give rise to several muscle types. The earliest event concerns the formation of the adaxial cells, which will later go on to form the slow muscles, whereas the rest of the somite will mainly produce fast muscle. Adaxial cells that are located at the apex of the somite will become muscle pioneers, cells strongly positive for Engrailed (*eng1*) [40]. Hh signaling from the midline induces both adaxial cells and muscle pioneers. As predicted by Hh injection experiments we find that both *myod *(Fig. 4A,B) and *eng1 *(Fig. 4C,D) [16,25,41] are strongly induced in *ptc1;ptc2 *mutants, indicating that these mutants mainly form slow muscle type fibers. Consistent with this somites appear to be elongated in the D/V direction and more cells appear to stack in the medial somite (See additional file 2). To gain further proof for a conversion of the myotome towards the slow muscle fate, we analyzed the expression of *prdm1*, known to be a key regulator for slow muscle cell differentiation [26]. In a wild type situation, *prdm1 *is expressed in the most posterior somites and spinal cord neurons (Fig. 4E,F). The expression of *prdm1 *is regulated by Hh signaling [27], which is confirmed by the fact that the expression level increases in the *ptc1 *mutant, and to an even higher level in the *ptc1;ptc2 *mutants. This strongly suggests that the differentiation between fast and slow muscle cell differentiation in the myotome is shifted towards slow muscle type fiber formation, possibly by upregulating *prdm1 *expression. Upregulation of *myoD *correlates with upregulation of *skeletal muscle alpha actin *(*acta1*) gene expression as a marker for muscle differentiation, and downregulation of Pax3/7 expression (See Additional files 1 and 2) a marker for the undifferentiated dermomyotomal cells [42].

Additionally, we also investigated whether the anteroposterior patterning of the somites was affected. Although *myod *is expressed in the posterior part of recently formed somites, the expansion of the adaxial domain of *myod *expression in *ptc1;ptc2 *mutants means that this marker cannot be used as A/P marker. Therefore, we analyzed *uncx4.1*, a marker whose expression is normally restricted to the posterior part of the somite, and surprisingly, we found that it was lost (Fig. 4G,H). In addition, expression of *fgf8*, which demarcates the anterior somite, was also lost (Fig. 4I,J), The loss of markers for both the anterior and posterior halves suggests that the somites have lost their polarity. Polarity defects are also corroborated by *myf5 *expression, which has a more complex expression pattern during somite maturation (Fig. 4K–N).

Since A/P polarity of the somites is likely to be determined during- and in fact may be necessary for, proper somite formation (epithelialization) we analyzed the expression of *deltaC *(*dlc*) (Fig. 4O,P), *her1 *(data not shown), and cellular morphology (See Additional file 2). In wild types, the expression of these genes shows oscillation in the presomitic mesoderm and is required for proper segmentation. These markers showed a normal expression pattern in presomitic mesoderm. Furthermore, epithelialization does occur, albeit less regularly. Furthermore *dlc *expression showed that although it is normal in presomitic mesoderm and during somite formation, it is not maintained in the posterior part of older, more anterior somites (Fig. 4O,P).

In an attempt to identify a link between increased Hh signaling and the loss of polarity markers, we analyzed a possible role for *prdm1 *in this process. Prdm1 can act as a transcriptional repressor, whose expression is controlled by Hh signaling [27]. Morpholino knockdown of *prdm1 *did not rescue the expression of *fgf8 *or *uncx4.1 *(data not shown), indicating that additional negative regulators are involved in the loss of polarity markers in the *ptc1;ptc2 *mutant somites. All these data suggest that the somites of *ptc1;ptc2 *mutants become apolar after somite boundaries have been established. To our knowledge, this is the first instance in which the process of somite formation has been genetically uncoupled from A/P patterning of the formed somite.

### Hedgehog signaling inhibits pectoral fin formation independent from RA signaling

**Figure 5:**
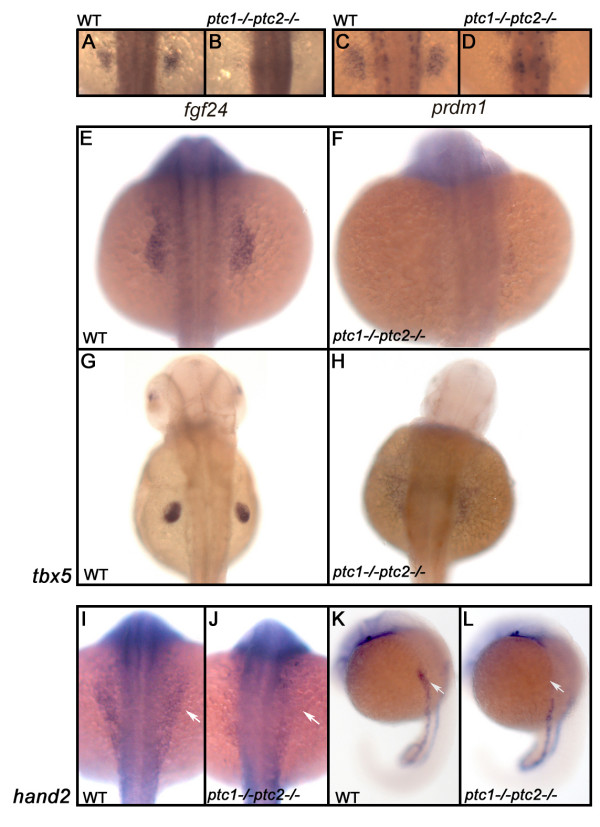
**Hh signaling has an early negative role in the induction of the fin field**. Expression analysis of *fgf24 *and *prdm1 *in a wild type embryo shows that these genes are restricted to the fin field at 26 hpf (A,C). *ptc1;ptc2 *mutants do not express these markers confirming that aberrant activation inhibits fin bud formation (B,D). To determine at which level Hh is inhibiting fin formation, the expression of *tbx5*, one of the earliest markers expressed in the finbud, was analyzed. At 20 hpf (E,F) *tbx5 *expression is lost in the presumptive finbud region (scale bar 100 μm). At 40 hpf (G,H), the fin bud has established and *tbx5 *expression is restricted to the pectoral fin in a wild type situation. However, in the *ptc1;ptc2 *double mutant a scattered low expression can be observed, showing that the pectoral fin bud is not formed. At the 10 ss, however, the initial expression domain of tbx5, encompassing heart and fin primordia is established. *hand2*, acting upstream of *tbx5 *is not expressed in the future pectoral fin area (I-L: white arrow), suggesting a very early negative role for Hh signaling in fin bud induction.

The Hh signaling pathway is known to regulate the formation and patterning of the vertebrate limb. However, Hh signaling is supposed to perform a rather late function in maintenance and outgrowth of the fin bud. Unexpectedly, the *ptc1;ptc2 *fin buds are completely lost. To investigate at which level Hh is acting on the induction of the fins, we investigated the expression pattern of several genes involved in fin development, like *fgf24 *and *prdm1*. Both genes were absent in the presumptive fin field (Fig. 5A–D). Additionally, the expression level of the transcription factor *tbx5*, one of the earliest markers for fin bud initiation was hardly detectable at 20 hpf, and a slight scattered expression was observed at 40 hpf (Fig. 5E–H). Initiation of *tbx5 *expression, labeling presumptive heart and pectoral fin primordia appeared normal, however (See Additional file 1). Hand2 (or dHAND) is another important factor in limb bud formation in zebrafish [43]. The expression of *tbx5 *and *hand2 *is mutually dependent since *hand2 *expression is reduced in *tbx5 *mutants and *tbx5 *expression is reduced in *hand2 *mutants [43,44]. We therefore tested whether we could see defects in *hand2 *expression. ISH at 20 hpf, shows the expected expression pattern of *hand2 *in posterior lateral mesoderm with a subtle difference in the future fin bud region where *hand2 *is absent (Fig. 5I–L). Published analysis of early stages of fin bud formation suggests that *tbx5 *is only mildly reduced in a *hand2 *mutant and fin buds are still formed [43]. The strong reduction of *tbx5 *in *ptc1;ptc2 *mutants suggests that although *hand2 *is reduced it is probably not the only causal factor. Since both *hand2 *and *tbx5 *are reduced we sought to identify defects in signals that might act upstream of these two genes. One factor that has been implicated in *tbx5 *expression is *wnt2b *[32], however no difference could be observed in *wnt2b *expression at 18 hpf (See Additional file 1). Finally, fin bud induction is known to be under direct control of RA signaling, as shown by the *neckless *(*nls*) mutant encoding *aldh1a2 *[45]. Furthermore, mouse Aldh1a2 -/- embryos neither express *tbx5 *nor *hand2 *[46]. Manipulation of RA signaling in the *nls *mutant, has shown that it is required before 10 somite stage (ss) [45] for development of the pectoral fin bud, as indicated by *dlx2 *expression. Additionally, RA signaling has also been shown to be necessary during early segmentation, when it is produced in the anterior somites, to induce the fin field [29], where it rescues *tbx5 *expression in the *neckless *mutant. In the *ptc1;ptc2 *mutant *aldh1a2 *expression, required for the production of RA, was not significantly altered at the 60% epiboly stage or at 18 somites in *ptc1;ptc2 *double mutants (data not shown). In an attempt to rescue the fin phenotype, we treated double mutant embryos with RA. Concentrations ranging from 10^-7 ^to 10^-6 ^M did not rescue the formation of the pectoral fins (n = 60 per concentration, including 3 to 4 *ptc1;ptc2 *mutants). However, the expected morphological defects in axis formation were obtained, showing that RA can signal in the *ptc1;ptc2 *mutants. We conclude that the Hh signaling pathway represses induction of the fin bud, and acts independent of RA signaling.

### Hedgehog signaling is inhibiting fin induction during segmentation

**Figure 6:**
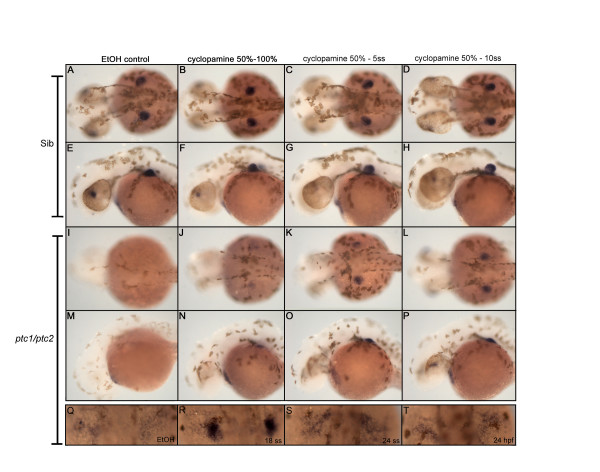
**Cyclopamine treatment determines time window where Hh signaling inhibits fin induction**. Treatment with 10 μM cyclopamine between 50% epiboly and the indicated developmental stages, identified the critical time window for Hh where it actively inhibits fin induction. From a dorsal (A,D, I-L) and lateral (E-H, M-P) view recruitment of *tbx5 *positive cells in the fin field can be slightly rescued when cyclopamine is administered between 50%- and 100% epiboly (J,N). However, *tbx5 *is more highly expressed when cyclopamine is administered between 50% epiboly and 5- and 10 somite stage (K,L,O,P). The constitutive activation of Hh signaling after removing cyclopamine, inhibits the outgrowth of the fin bud, clearly visible from a lateral view (M-P). Inhibiting Hh signaling in *ptc1;ptc2 *double mutants from the 18 ss till 40 hpf, rescues a restricted expression of *tbx5*, which is not, observed when cyclopamine is administered at 24 ss or 24 hpf (Q-T). These data show that Hh signaling inhibits fin induction during late gastrulation and the segmentation stage.

Since the expression of *wnt2b *is not affected in the *ptc1;ptc2 *mutants and exogenous RA treatment is unable to rescue the fin phenotype, we tried to determine in which time window Hh is preventing fin field induction. Therefore, we treated 90 embryos, obtained from a *ptc1*^+/-^;*ptc2*^+/- ^incross, with 10 μM cyclopamine between 50% epiboly and different developmental stages. This concentration of cyclopamine does not severely affect wild type siblings. By using this rather low concentration we do not affect the later role for Hh in outgrowing of the fin bud, but specifically focus on its supposed early role in fin bud induction. As a readout for a possible rescue, we examined *tbx5 *expression at 40 hpf, showing a clearly distinct finbud in the wild types (Fig. 6A–D,E–H) and an absence of *tbx5 *in the *ptc1;ptc2 *mutant (Fig. 6I,M). Transient inhibition of Hh by cyclopamine between 50% and 100% epiboly (Fig. 6B,F,J,N) did not re-establish a localized *tbx5 *expression in the presumptive fin field. However, low *tbx5 *expression could be detected, in contrast to the untreated *ptc1;ptc2 *mutant (Fig. 6I,M). Localized expression of *tbx5 *was detected when Hh was inhibited between 50% epiboly and 5 ss (Fig. 6K,O). This can be enhanced when *ptc1;ptc2 *mutants are treated with cyclopamine between 50% and the 10 ss (Fig. 6L,P). Thus inhibiting Hh signaling during early segmentation stages clearly rescued *tbx5 *expression in the fin field. However, it is not clear how quickly the cyclopamine is washed out. Furthermore no obvious outgrowth of the pectoral fin bud was observed in these embryos at 40 hpf, which indicates that high levels of Hh signaling can still inhibit pectoral fin outgrowth after the 10 ss in the *ptc1;ptc2 *mutant. To further investigate the time window in which Hh is negatively regulating fin induction, we treated embryos with 10 μM cyclopamine from 12 ss, 18 ss, 24 ss and 24 hpf towards 40 hpf (Fig. 6Q–T). Restricted expression of *tbx5 *can only be rescued when Hh activity is inhibited starting from the 12 (data not shown) or 18 somite stage (Fig. 6R). Adding cyclopamine at 24 ss or 24 hpf results in a scattered *tbx5 *expression (Fig. 6S,T) which is nearly similar to the untreated *ptc1;ptc2 *mutants (Fig. 6Q). These experiments suggest that Hh signaling is inhibiting recruitment of *tbx5 *positive cells during the segmentation period (from 100% epiboly until 24 somite stage). During this time, the closest source of potential Hh signaling is at the midline. The normal fin primordium is located approximately 130-150μm, this is somewhat further than the estimated active range of midline hedgehog signaling (in chick neural tube approximately 100μm [47]) and would suggest such signals would normally not  occur in the fin region.

## Discussion

### Isolation and characterization of a Patched1 mutant

Our previous research has shown that the Hh signaling pathway is strictly controlled in zebrafish to prevent overactivation, since the concurrent loss of Sufu, Hip and Ptc2 does not result in the severe morphological defects [14], that have been described for Hh overactivation. We previously hypothesized that a combined loss of both Patched proteins should result in a constitutive activation of the Hh pathway. Contrary to Ptc2, there was no zebrafish Ptc1 mutant available to confirm this idea. Here we report the identification of a zebrafish Ptc1 mutant via a reverse genetic approach. The identified splice donor mutation, results in the insertion of intron 10 consisting of 81 base pairs into the transcript, enlarging the second extracellular loop of the protein by 27 amino acids and the inserted amino acid sequence has no homology to known proteins or domains. Previous reports have described that the first and fourth extracellular loops are required to bind Hh [47]. There are two likely explanations for the observed effect. Firstly, the modified protein is not inserted correctly in the membrane, and can therefore not exert its normal function. An alternative possibility is that the extended loop mimics the effect that Hh binding normally has, or that it somehow affects the sterol-sensing domain. This scenario might result in a correctly localized protein, which would however, lose its repressive capacity on Smoothened. Although the consequence of this insertion on a protein level is hard to predict, the Hh pathway is clearly constitutively activated, since *ptc1, nkx2.2a *and *gli1 *expression are severely increased in *ptc1*^*hu1602 *^mutants.

Zebrafish mutants homozygous for *ptc1*^*hu1602*^, show a somite defect, as expected from Hh overexpression experiments, where the angle of the somite becomes more obtuse. However, the strength of the phenotype is surprisingly mild, since mouse data suggest that Ptc1 is the main inhibitor of Hh signaling. In mice, *ptc1 *knockouts are embryonic lethal [48] and *ptc2 *mutants are viable without obvious defects [49], indicating that Ptc1 is the major regulator in mammals. This could indicate a functional shift between zebrafish and the mouse homologues of Ptc. This shift may also be reflected by the fact that the comparisons between the mouse and fish genes does not result in a clear one-to-one relationship between the Ptc1 and Ptc2 homologs [50].

### *ptc1;ptc2 *double mutants show the consequences of constitutive activation of Hh signaling

The *ptc1 *mutant enabled us to confirm our hypothesis that in zebrafish Ptc1 is the key negative regulator of the Hh pathway, conferring a stronger phenotype than the triple mutant made with Sufu, Hip and Ptc2. The *ptc1;ptc2 *double mutants showed the expected flat somite phenotype, which is the classical effect of Hh overexpression. Additionally, mutants homozygous for both the *ptc1*^*hu1602 *^and *uki(hip)*^*hu418B *^mutations also resulted in flattened somites confirming the idea that *ptc1 *expression is induced above a certain activity, thereby preventing the pathway from further activation (data not shown). This is a likely scenario since subtle increases in *ptc1 *expression could be observed in *ptc2 *and *hip *mutants [14], confirming the idea that Ptc1 has a pivotal role in controlling the activity of the pathway. The constitutive activation of the pathway in *ptc1;ptc2 *double mutants results in similar phenotypes as described after injection of *shh *or dnPKA, concerning the optic stalk versus cup differentiation in the developing eye, patterning of the floor plate and differentiation of fast and slow muscle cell types, thereby validating these mutants as a model system to study the consequences of aberrantly activated Hh signaling.

### Inhibition of the Hh signaling pathway is necessary for the establishment of somite polarity

Somite A/P information has previously been shown to be necessary for proper segmentation [13,51]. In the zebrafish mutants *fused somites *(*fss*) [13], anterior information is lost and the complete somite is posteriorized [51], resulting in the lack of somite boundary formation. When anterior and posterior polarity genes are expressed throughout the complete somite, segmentation is disturbed. This has been shown for the *beamter *(*bea*), *deadly seven *(*des*) and *after eight *(*aei*) mutants [13], which do only form the first four, seven and eight somites respectively, as a consequence of mutated components of the Notch signaling pathway [52,53]. The A/P axis is thought to be established in the presomitic mesoderm, and is thought to be required for morphological boundary formation [51].

The *ptc1;ptc2 *mutants show that A/P patterning in the presomitic mesoderm/nascent somite can be separated from maintenance of A/P patterning in existing somites. We analyzed expression of *her1 *and *dlc *in the presomitic mesoderm and found no defects, indicating that the oscillations in their expression occur normally. This is confirmed by a normal morphological progression of somitogenesis in double mutants. From the known role of Hh signaling in paraxial mesoderm, and *myod *labeling experiments, we expect that the paraxial mesoderm is completely induced to become adaxial. We conclude that adaxial cells are capable of executing the oscillations required for segmentation and are capable of forming somite boundaries. However, analysis of *dlc *has shown that the somites are not able to maintain the A/P pattern in the somites. Surprisingly, such somites do not show a default A or P identity, rather they appear to be apolar (loss of *fgf8*/anterior; *uncx4.1*/posterior).

Since the Hh signaling pathway leads to activation of transcription via the Gli genes, repression of the former genes has to occur via intermediate repressors. *prdm1 *was tested as an good candidate, but morpholino-knockdown of this gene could not rescue *fgf8 *or *uncx4.1 *expression, indicating that additional repressors are induced. Since we have tested only a limited set of markers we cannot exclude that the somites have some currently undetectable A/P polarity.

Most markers that show A/P differences are not expressed in the adaxial cells or are expressed in all adaxial cells without displaying A/P differences. Thus it might be assumed that polarity is lost because adaxial cells do not participate in this patterning, and Hh signaling from the midline induces adaxial fates. Loss of *hh *signaling has not been shown to affect somite A/P patterning, thus such a interference model appears attractive. Even though the effect on *myf5 *expression suggests that A/P is directly by the *hh *signal, our data currently cannot exclude an indirect model where premature muscle differentiation prevents expression of such markers.

Somite A/P pattern has been reported to guide patterning of the motoneurons of the neural tube [54]. In mutants *ptc1;ptc2 *neural tube patterning itself is affected by ectopic Hh signaling, thus in order to study this tissue specific knockouts are required. Nevertheless, we find defects that are at least consistent with defects in A/P patterning (See Additional file 2).

### Hedgehog is a negative regulator of pectoral fin formation

One of the most surprising phenotypes of the *ptc1;ptc2 *double mutant is the absence of the pectoral fins. Thus far, only a positive role for Shh signaling in limb development has been described, and this function is supposed to be later in development after the establishment of the limb bud [55-58]. Indeed, zebrafish mutants that abolish Hh signaling, like *syu *or *smu*, do have fin buds, but these fail to grow out [35,59,60]. Although Shh is required late in limb formation, Gli3, a downstream component of the Hh pathway, may act earlier. This has been shown convincingly by several reports [61-63]. The results indicate that the repressor form of Gli3 (Gli3R) prevents expression of *hand2 *in the anterior limb bud, thereby prepatterning this structure. According to this model activation of Hh signaling should lead to processing of Gli3R to Gli3Act and *hand2 *should become expressed in the anterior limb bud. This leads to expansion of posterior genes and ectopic *shh *expression. Morphologically it should lead to polydactylous limbs rather than absence of limbs, similar to what has been observed in mouse Gli3 deficient limbs. This is in fact the opposite of what we find in *ptc1;ptc2 *double mutants. We find that *hand2 *is suppressed and that *shh *expression is lost in the prospective fin bud. One possible explanation for this discrepancy is that there are evolutionary differences between fish and more advanced vertebrates. Therefore, it will be interesting to create a mouse *ptc1;ptc2 *double mutant to test whether the findings from zebrafish hold up in mammals. Recently, a mouse *ptc2 *mutant has been generated [49], but since the Ptc1 mutant [64] is embryonic lethal, more sophisticated inducible mutants should be generated to test whether our finding on fin induction are conserved in mammals.

Retinoic acid may have an early role in establishing the fin bud territory, since RA has been found to affect *tbx5 *and *hand2 *expression. During segmentation, RA formed in the first few somites is necessary to induce fin induction with a critical time window between gastrulation and 12 hpf [29]. We have not found any evidence that the effect of Hh acts through a RA signal. The expression of *aldh1a2 *is not altered by Hh activation and exogenous RA does not rescue pectoral fin formation. Furthermore, *wnt2b* acts downstream of RA to establish the pectoral fin territory, and its expression is also unaffected in the *ptc1;ptc2 *mutant. Our results suggest that Hh signaling may have a very early negative effect on the establishment of the fin field in the zebrafish.

At the 10 somite stage when *tbx5 *expression is just established [65,66] and normal in double mutants, we find *ptc2 *expression both in the somites and lateral plate, whereas *ptc1 *is only detectable in the somites, however, in *ptc1;ptc2 *double mutants *ptc1 *becomes expressed more laterally as well (See Additional file 1). Thus it is possible that Patched proteins function around this stage in the fin primordium to maintain *tbx5 *expression. This does not exactly fit with our data that cyclopamine rescues already at the 50%- 5 somite stage. However, rapid development, in combination with perdurance of the cyclopamine after wash-out could account for this. It might be possible that Hh signals from the midline could put a limit to the fin bud territory at the medial side. However such a model would imply that loss of Hh signaling should lead to an increase in the *tbx5 *expression domain towards the midline, which has not been reported [65]. To accommodate for this, such a model would require a second unknown signal that inhibits expansion in parallel to Hh.

An alternative explanation would be that a subset of cells in the somites is transformed into adaxial cells/slow muscle, or intermediate mesoderm could be affected. As a result of this transformation a cell type may be lost that is capable of inducing the pectoral fin primordia, or gain a signal that blocks formation of these primordia, creating a neomorphic phenotype. Indeed, the *lbx1 *positive population of premigratory limb muscle cells is lost in *ptc1;ptc2 *mutants (See Additional file 1), but there is currently no evidence that these particular cells are required to form a limb bud in mice or fish [67,68] Again this model would require postulation of a second unknown signal. In the case of a positive signal, it would be required parallel to the "RA/Wnt2b" signal. Elegant experiments from Gibert et al. [29], suggest that there is no second positive signal from the somites, since application of RA in embryos in which the paraxial mesoderm is genetically ablated can induce early fin markers.

We currently favor the first model where Hh signaling can inhibit *tbx5 *directly in the fin primordia but that this is normally redundant and may help to make pectoral fin development more robust. Transplantation experiments may help to resolve where *ptc1 *and *ptc2 *exert their inhibitory function through the somites or directly in fin primordium, but such experiments will be challenging due to the low frequency of *ptc1;ptc2 *double mutants, and the inability to identify such animals at the stage these experiments are normally performed.

## Conclusion

Altogether, this report describes the identification of two zebrafish *ptc1 *mutants and two novel and unexpected roles for Hh signaling during development, as observed in the *ptc1;ptc2 *mutant. Using this model system, we are now able to study the consequences of constitutive activation of Hh signaling during vertebrate development, in a stable genetic manner, which will be important for fully understanding the role of Hh during development and disease.

## Methods

### Zebrafish lines and maintenance

Zebrafish were maintained and staged according to standard protocols [69]. Zebrafish lines used: *ptc1*^*hu1602*^, *lep*^*tj222*^, *blw *^*tc294z*^.

### Screening Mutation library

An ENU induced mutation library was screened for Ptc1 mutants, analyzing an amplicons covering exon 9–12, using the TILLING protocol [36]. Primer sequences can be obtained upon request.

### Morpholino/mRNA injections

MOs for Ptc1 and LynGFP mRNA (a kind gift of R. Kim) were injected at the 1-cell stage according to [25] in a range from 0.5 to 3 ng for the MO and approximately 1 nl of 25 ng/μl for LynGFP. Prdm1 MOs were injected according to Baxendale et al, 2004 [26].

### Whole mount in situ hybridizations and immunohistochemistry

ISH experiments were performed as described in Thisse et al, 1993 [70]. The following probes where generated according to the indicated articles: *ptc1 *[15], *fgf8 *[71], *myod *[72], *tbx5 *[31], *hand2 *[42], *uncx4.1 *[73], *eng1 *[41], *dlc *[52]), *dlx2 *[74], *gli1 *[6], *foxa1 *[75]*, ptc2 *[50], *shh *[76]*, islet1 *[77]*, acta1 *(a gift of S. Baxendale)*, pax2, pax6 *[78]*, lbx1 *[79]. The *prdm1 *and *nkx2.2a *probes were generated from PCR product, using T3-tailed primers and subsequent transcription with T3-RNA polymerase. Whole mount immunohistochemistry was performed according to standard protocols [80] we used a pan-islet monoclonal antibody 39.4D5 (Developmental Studies Hybridoma bank) and monoclonal DP312 [81] to label pax3/7 cells [42] Pax3/7 was detected using Cy3 conjugated donkey anti-mouse secondary antibody (Jackson Immuno Research), mounted in Vectashield+DAPI (Vector Labs) and viewed under a Zeiss LSM510 confocal microscope.

### Retinoic acid treatment

Progeny of *ptc1*^*hu1602*^/+ ; *lep*(*ptc2*)+/- was treated with *all-trans *RA as described before [82]. Concentrations were ranging from 10^-6 ^to 10^-7 ^M, diluted from a 10^-2 ^M stock in DMSO. Embryos were treated and analyzed from 4 hpf till 48 hpf. Genotypes were determined subsequently on the complete clutch to confirm the presence of *ptc1;ptc2 *double mutants.

### Cyclopamine treatment

Cyclopamine was dissolved in 96% EtOH to a concentration of 10 mM. Zebrafish embryos were treated from 5.5 hpf with varying concentrations of cyclopamine and controls with an equal volume of 96% EtOH.

## Abbreviations

A/P: anterior/posterior; Cos2: Costal2; Hh: Hedgehog; Hip: Hedgehog interacting protein; hpf: hours post fertilization; ISH: in situ hybridization; LPM: lateral plate mesoderm; MO: Morpholino; RA: retinoic acid; RPE: retinal pigmented epithelium; ss: somite stage; Sufu: Suppressor of Fused; Ptc: Patched; MiP middle primary neuron, CaP caudal primary neuron.

## Authors' contributions

MJK conducted most of the experiments and wrote the article. MJB and EG performed experiments. FJE conceived of the study, and participated in its design and coordination and helped to draft the manuscript. All authors read and approved to the final manuscript.

## Supplementary Material

Additional file 1 A,B,C,D *ptc1 *expression in wild-type (A), *ptc1/+ *(B), *ptc1 *mutant (C) and *ptc1;ptc2 *mutant (D) stained in a single reaction 19 somite stage; no significant increase is detectable in *ptc2 *mutants at this stage. E,G,I,K,M,O,Q,S,V,W,X) wild type embryos; F,H,J,L,N,P,R,T,U,Y) *ptc1;ptc2 *mutants. E,F) 21 somite stage; *Gli *expression is increased *ptc1;ptc2 *mutants. G,H) *foxa1 *expression labels medial and lateral floor plate and is expanded in *ptc1;ptc2 *mutants, *shh *a medial floor plate marker, remains unchanged (inset). I,J,K,L) 18 somite stage; *pax2 *labeling of the optic stalk in wild type (I) is expanded in *ptc1;ptc2 *mutants (J). At the same time *pax6 *expression in the optic cup is severly reduced in *ptc1;ptc2 *mutants (K,L). M,N) 12 somite stage, flat mount, dorsal view; *skeletal muscle alpha actin1*, a marker for muscle differentiation is increased in *ptc1;ptc2 *mutants. O,P) 20 somite stage, ventral part of anterior somites. Presumptive migratory myoblasts that will form fin muscle express *lbx1*. Expression of this gene is lost in *ptc1;ptc2 *mutants. Q,R) Oblique view on *wnt2b *expression focusing on region between lateral plate and ventral somites 21 somite stage. This gene is required for fin bud formation and is still expressed in *ptc1;ptc2 *mutants. S,T) 10 somite stage, flat mount, dorsal view. Initial expression of *tbx5 *in the -then continuous- heart and pectoral fin primordium is unaltered in *ptc1;ptc2 *mutants (arrowheads). Double mutants lack *tbx5 *expression in the optic cup (star). U,V,W,X,Y) 10 somite stage flat mount dorsal view U,V) *ptc1;ptc2 *mutants and wild-type show tbx5 labeling at the edge of the lateral plate mesoderm, *ptc2 *is also expressed in this region (W). *ptc1 *expression is normally only detectable in the somites strongly in the adaxial cells and weakly in the lateral somite. However, *ptc1 *is upregulated throughout the entire embryo in *ptc1;ptc2 *mutants, showing that it can respond to Hh signaling in lateral plate mesoderm. Note: X and Y were stained for equal time is the same reaction. Click here for file

Additional file 2 A,C,E,G,J) wild type embryo; B,D,F,H,I,K) *ptc1;ptc2 *mutants. A,B) lynGFP membrane label showing most recent fully formed somite, 14 somite stage. Double mutant somites still form an epithelium but irregularities in the epithelial somite are more frequent. C,D) DAPI nuclear stain showing essentially the same result as A,B. E,F) LynGFP labeling outlining cells of differentiating somites in a 14 somite wild-type and *ptc1;ptc2 *mutant embryo, medial optical section through somite 10 and 9+10, respectively. Somites have lost their clear V shape and the number of elongated adaxial cells appears increased. G,H,I) Double labeling showing anti-Islet (brown) and *islet1 *expression (20 ss). Brown cells express only Islet 2 and are Caudal Primary (CaP) neurons, blue/brown cells express *islet 1 *(and possibly 2) and are Middle Primary neurons (MiP). In wild-type (G) brown CaPs are located in the middle of each segment whereas the blue/brown MiPs are close to the somite boundary (drawn-in in G for clarity) [54]. In a *ptc1;ptc2 *mutant background (H,I) mistakes in this order are very frequent, for instance, blue and brown cells close to the posterior of the segment, or the exact mirror image of that (H, arrows). In (I) a brown cell can be seen in the position where a blue cell would be expected (arrowhead). J,K) Z-projection of Pax3/7 labeling (red) and nuclear DAPI stain (blue) on posterior 5–6 somites of 20 somite stage embryos showing the loss of all pax3/7 staining in the somites, and a reduction of the number of pax3/7 positive nuclei in the dorsal neural tube in *ptc1;ptc2 *double mutants. Click here for file

## References

[B1] McMahon AP, Ingham PW, Tabin CJ (2003). Developmental roles and clinical significance of hedgehog signaling. Curr Top Dev Biol.

[B2] Lum L, Beachy PA (2004). The Hedgehog response network: sensors, switches, and routers. Science.

[B3] Huangfu D, Anderson KV (2006). Signaling from Smo to Ci/Gli: conservation and divergence of Hedgehog pathways from Drosophila to vertebrates. Development.

[B4] Schauerte HE, van Eeden FJ, Fricke C, Odenthal J, Strahle U, Haffter P (1998). Sonic hedgehog is not required for the induction of medial floor plate cells in the zebrafish. Development.

[B5] Barresi MJ, Stickney HL, Devoto SH (2000). The zebrafish slow-muscle-omitted gene product is required for Hedgehog signal transduction and the development of slow muscle identity. Development.

[B6] Karlstrom RO, Tyurina OV, Kawakami A, Nishioka N, Talbot WS, Sasaki H, Schier AF (2003). Genetic analysis of zebrafish gli1 and gli2 reveals divergent requirements for gli genes in vertebrate development. Development.

[B7] Karlstrom RO, Talbot WS, Schier AF (1999). Comparative synteny cloning of zebrafish you-too: mutations in the Hedgehog target gli2 affect ventral forebrain patterning. Genes Dev.

[B8] Nakano Y, Kim HR, Kawakami A, Roy S, Schier AF, Ingham PW (2004). Inactivation of dispatched 1 by the chameleon mutation disrupts Hedgehog signalling in the zebrafish embryo. Dev Biol.

[B9] Wolff C, Roy S, Lewis KE, Schauerte H, Joerg-Rauch G, Kirn A, Weiler C, Geisler R, Haffter P, Ingham PW (2004). iguana encodes a novel zinc-finger protein with coiled-coil domains essential for Hedgehog signal transduction in the zebrafish embryo. Genes Dev.

[B10] Sekimizu K, Nishioka N, Sasaki H, Takeda H, Karlstrom RO, Kawakami A (2004). The zebrafish iguana locus encodes Dzip1, a novel zinc-finger protein required for proper regulation of Hedgehog signaling. Development.

[B11] Woods IG, Talbot WS (2005). The you gene encodes an EGF-CUB protein essential for Hedgehog signaling in zebrafish. PLoS Biol.

[B12] Kawakami A, Nojima Y, Toyoda A, Takahoko M, Satoh M, Tanaka H, Wada H, Masai I, Terasaki H, Sakaki Y, Takeda H, Okamoto H (2005). The zebrafish-secreted matrix protein you/scube2 is implicated in long-range regulation of hedgehog signaling. Curr Biol.

[B13] van Eeden FJ, Granato M, Schach U, Brand M, Furutani-Seiki M, Haffter P, Hammerschmidt M, Heisenberg CP, Jiang YJ, Kane DA, Kelsh RN, Mullins MC, Odenthal J, Warga RM, Allende ML, Weinberg ES, Nusslein-Volhard C (1996). Mutations affecting somite formation and patterning in the zebrafish, Danio rerio. Development.

[B14] Koudijs MJ, den Broeder MJ, Keijser A, Wienholds E, Houwing S, van Rooijen EM, Geisler R, van Eeden FJ (2005). The Zebrafish Mutants dre, uki, and lep Encode Negative Regulators of the Hedgehog Signaling Pathway. PLoS Genet.

[B15] Concordet JP, Lewis KE, Moore JW, Goodrich LV, Johnson RL, Scott MP, Ingham PW (1996). Spatial regulation of a zebrafish patched homologue reflects the roles of sonic hedgehog and protein kinase A in neural tube and somite patterning. Development.

[B16] Hammerschmidt M, Bitgood MJ, McMahon AP (1996). Protein kinase A is a common negative regulator of Hedgehog signaling in the vertebrate embryo. Genes Dev.

[B17] Ekker SC, Ungar AR, Greenstein P, von Kessler DP, Porter JA, Moon RT, Beachy PA (1995). Patterning activities of vertebrate hedgehog proteins in the developing eye and brain. Curr Biol.

[B18] Barth KA, Wilson SW (1995). Expression of zebrafish nk2.2 is influenced by sonic hedgehog/vertebrate hedgehog-1 and demarcates a zone of neuronal differentiation in the embryonic forebrain. Development.

[B19] Wilson L, Maden M (2005). The mechanisms of dorsoventral patterning in the vertebrate neural tube. Dev Biol.

[B20] Ingham PW, Kim HR (2005). Hedgehog signalling and the specification of muscle cell identity in the zebrafish embryo. Exp Cell Res.

[B21] Stickney HL, Barresi MJ, Devoto SH (2000). Somite development in zebrafish. Dev Dyn.

[B22] Blagden CS, Currie PD, Ingham PW, Hughes SM (1997). Notochord induction of zebrafish slow muscle mediated by Sonic hedgehog. Genes Dev.

[B23] Currie PD, Ingham PW (1996). Induction of a specific muscle cell type by a hedgehog-like protein in zebrafish. Nature.

[B24] Du SJ, Devoto SH, Westerfield M, Moon RT (1997). Positive and negative regulation of muscle cell identity by members of the hedgehog and TGF-beta gene families. J Cell Biol.

[B25] Wolff C, Roy S, Ingham PW (2003). Multiple muscle cell identities induced by distinct levels and timing of hedgehog activity in the zebrafish embryo. Curr Biol.

[B26] Baxendale S, Davison C, Muxworthy C, Wolff C, Ingham PW, Roy S (2004). The B-cell maturation factor Blimp-1 specifies vertebrate slow-twitch muscle fiber identity in response to Hedgehog signaling. Nat Genet.

[B27] Roy S, Wolff C, Ingham PW (2001). The u-boot mutation identifies a Hedgehog-regulated myogenic switch for fiber-type diversification in the zebrafish embryo. Genes Dev.

[B28] Lin Y, Wong K, Calame K (1997). Repression of c-myc transcription by Blimp-1, an inducer of terminal B cell differentiation. Science.

[B29] Gibert Y, Gajewski A, Meyer A, Begemann G (2006). Induction and prepatterning of the zebrafish pectoral fin bud requires axial retinoic acid signaling. Development.

[B30] Mercader N, Fischer S, Neumann CJ (2006). Prdm1 acts downstream of a sequential RA, Wnt and Fgf signaling cascade during zebrafish forelimb induction. Development.

[B31] Tamura K, Yonei-Tamura S, Belmonte JC (1999). Differential expression of Tbx4 and Tbx5 in Zebrafish fin buds. Mech Dev.

[B32] Ng JK, Kawakami Y, Buscher D, Raya A, Itoh T, Koth CM, Rodriguez Esteban C, Rodriguez-Leon J, Garrity DM, Fishman MC, Izpisua Belmonte JC (2002). The limb identity gene Tbx5 promotes limb initiation by interacting with Wnt2b and Fgf10. Development.

[B33] Fischer S, Draper BW, Neumann CJ (2003). The zebrafish fgf24 mutant identifies an additional level of Fgf signaling involved in vertebrate forelimb initiation. Development.

[B34] Roelink H, Augsburger A, Heemskerk J, Korzh V, Norlin S, Ruiz i Altaba A, Tanabe Y, Placzek M, Edlund T, Jessell TM (1994). Floor plate and motor neuron induction by vhh-1, a vertebrate homolog of hedgehog expressed by the notochord. Cell.

[B35] Neumann CJ, Grandel H, Gaffield W, Schulte-Merker S, Nusslein-Volhard C (1999). Transient establishment of anteroposterior polarity in the zebrafish pectoral fin bud in the absence of sonic hedgehog activity. Development.

[B36] Wienholds E, van Eeden F, Kosters M, Mudde J, Plasterk RH, Cuppen E (2003). Efficient target-selected mutagenesis in zebrafish. Genome Res.

[B37] Karlstrom RO, Trowe T, Klostermann S, Baier H, Brand M, Crawford AD, Grunewald B, Haffter P, Hoffmann H, Meyer SU, Muller BK, Richter S, van Eeden FJ, Nusslein-Volhard C, Bonhoeffer F (1996). Zebrafish mutations affecting retinotectal axon pathfinding. Development.

[B38] Macdonald R, Barth KA, Xu Q, Holder N, Mikkola I, Wilson SW (1995). Midline signalling is required for Pax gene regulation and patterning of the eyes. Development.

[B39] Odenthal J, van Eeden FJ, Haffter P, Ingham PW, Nusslein-Volhard C (2000). Two distinct cell populations in the floor plate of the zebrafish are induced by different pathways. Dev Biol.

[B40] Hatta K, Bremiller R, Westerfield M, Kimmel CB (1991). Diversity of expression of engrailed-like antigens in zebrafish. Development.

[B41] Ekker M, Wegner J, Akimenko MA, Westerfield M (1992). Coordinate embryonic expression of three zebrafish engrailed genes. Development.

[B42] Hammond CL, Hinits Y, Osborn DP, Minchin JE, Tettamanti G, Hughes SM (2007). Signals and myogenic regulatory factors restrict pax3 and pax7 expression to dermomyotome-like tissue in zebrafish. Dev Biol.

[B43] Yelon D, Ticho B, Halpern ME, Ruvinsky I, Ho RK, Silver LM, Stainier DY (2000). The bHLH transcription factor hand2 plays parallel roles in zebrafish heart and pectoral fin development. Development.

[B44] Garrity DM, Childs S, Fishman MC (2002). The heartstrings mutation in zebrafish causes heart/fin Tbx5 deficiency syndrome. Development.

[B45] Grandel H, Lun K, Rauch GJ, Rhinn M, Piotrowski T, Houart C, Sordino P, Kuchler AM, Schulte-Merker S, Geisler R, Holder N, Wilson SW, Brand M (2002). Retinoic acid signalling in the zebrafish embryo is necessary during pre-segmentation stages to pattern the anterior-posterior axis of the CNS and to induce a pectoral fin bud. Development.

[B46] Mic FA, Sirbu IO, Duester G (2004). Retinoic acid synthesis controlled by Raldh2 is required early for limb bud initiation and then later as a proximodistal signal during apical ectodermal ridge formation. J Biol Chem.

[B47] Marigo V, Davey RA, Zuo Y, Cunningham JM, Tabin CJ (1996). Biochemical evidence that patched is the Hedgehog receptor. Nature.

[B48] Milenkovic L, Goodrich LV, Higgins KM, Scott MP (1999). Mouse patched1 controls body size determination and limb patterning. Development.

[B49] Lee Y, Miller HL, Russell HR, Boyd K, Curran T, McKinnon PJ (2006). Patched2 modulates tumorigenesis in patched1 heterozygous mice. Cancer Res.

[B50] Lewis KE, Concordet JP, Ingham PW (1999). Characterisation of a second patched gene in the zebrafish Danio rerio and the differential response of patched genes to Hedgehog signalling. Dev Biol.

[B51] Durbin L, Sordino P, Barrios A, Gering M, Thisse C, Thisse B, Brennan C, Green A, Wilson S, Holder N (2000). Anteroposterior patterning is required within segments for somite boundary formation in developing zebrafish. Development.

[B52] Holley SA, Geisler R, Nusslein-Volhard C (2000). Control of her1 expression during zebrafish somitogenesis by a delta-dependent oscillator and an independent wave-front activity. Genes Dev.

[B53] Julich D, Hwee Lim C, Round J, Nicolaije C, Schroeder J, Davies A, Geisler R, Lewis J, Jiang YJ, Holley SA (2005). beamter/deltaC and the role of Notch ligands in the zebrafish somite segmentation, hindbrain neurogenesis and hypochord differentiation. Dev Biol.

[B54] Lewis KE, Eisen JS (2004). Paraxial mesoderm specifies zebrafish primary motoneuron subtype identity. Development.

[B55] Riddle RD, Johnson RL, Laufer E, Tabin C (1993). Sonic hedgehog mediates the polarizing activity of the ZPA. Cell.

[B56] Chiang C, Litingtung Y, Lee E, Young KE, Corden JL, Westphal H, Beachy PA (1996). Cyclopia and defective axial patterning in mice lacking Sonic hedgehog gene function. Nature.

[B57] Chiang C, Litingtung Y, Harris MP, Simandl BK, Li Y, Beachy PA, Fallon JF (2001). Manifestation of the limb prepattern: limb development in the absence of sonic hedgehog function. Dev Biol.

[B58] Kraus P, Fraidenraich D, Loomis CA (2001). Some distal limb structures develop in mice lacking Sonic hedgehog signaling. Mech Dev.

[B59] Varga ZM, Amores A, Lewis KE, Yan YL, Postlethwait JH, Eisen JS, Westerfield M (2001). Zebrafish smoothened functions in ventral neural tube specification and axon tract formation. Development.

[B60] Chen W, Burgess S, Hopkins N (2001). Analysis of the zebrafish smoothened mutant reveals conserved and divergent functions of hedgehog activity. Development.

[B61] Wang B, Fallon JF, Beachy PA (2000). Hedgehog-regulated processing of Gli3 produces an anterior/posterior repressor gradient in the developing vertebrate limb. Cell.

[B62] te Welscher P, Fernandez-Teran M, Ros MA, Zeller R (2002). Mutual genetic antagonism involving GLI3 and dHAND prepatterns the vertebrate limb bud mesenchyme prior to SHH signaling. Genes Dev.

[B63] te Welscher P, Zuniga A, Kuijper S, Drenth T, Goedemans HJ, Meijlink F, Zeller R (2002). Progression of vertebrate limb development through SHH-mediated counteraction of GLI3. Science.

[B64] Jeong J, McMahon AP (2005). Growth and pattern of the mammalian neural tube are governed by partially overlapping feedback activities of the hedgehog antagonists patched 1 and Hhip1. Development.

[B65] Begemann G, Ingham PW (2000). Developmental regulation of Tbx5 in zebrafish embryogenesis. Mech Dev.

[B66] Ahn DG, Kourakis MJ, Rohde LA, Silver LM, Ho RK (2002). T-box gene tbx5 is essential for formation of the pectoral limb bud. Nature.

[B67] Haines L, Neyt C, Gautier P, Keenan DG, Bryson-Richardson RJ, Hollway GE, Cole NJ, Currie PD (2004). Met and Hgf signaling controls hypaxial muscle and lateral line development in the zebrafish. Development.

[B68] Gross MK, Moran-Rivard L, Velasquez T, Nakatsu MN, Jagla K, Goulding M (2000). Lbx1 is required for muscle precursor migration along a lateral pathway into the limb. Development.

[B69] Kimmel CB, Ballard WW, Kimmel SR, Ullmann B, Schilling TF (1995). Stages of embryonic development of the zebrafish. Dev Dyn.

[B70] Thisse C, Thisse B, Schilling TF, Postlethwait JH (1993). Structure of the zebrafish snail1 gene and its expression in wild-type, spadetail and no tail mutant embryos. Development.

[B71] Reifers F, Bohli H, Walsh EC, Crossley PH, Stainier DY, Brand M (1998). Fgf8 is mutated in zebrafish acerebellar (ace) mutants and is required for maintenance of midbrain-hindbrain boundary development and somitogenesis. Development.

[B72] Weinberg ES, Allende ML, Kelly CS, Abdelhamid A, Murakami T, Andermann P, Doerre OG, Grunwald DJ, Riggleman B (1996). Developmental regulation of zebrafish MyoD in wild-type, no tail and spadetail embryos. Development.

[B73] Kawakami Y, Raya A, Raya RM, Rodriguez-Esteban C, Belmonte JC (2005). Retinoic acid signalling links left-right asymmetric patterning and bilaterally symmetric somitogenesis in the zebrafish embryo. Nature.

[B74] Akimenko MA, Ekker M, Wegner J, Lin W, Westerfield M (1994). Combinatorial expression of three zebrafish genes related to distal-less: part of a homeobox gene code for the head. J Neurosci.

[B75] Odenthal J, Nusslein-Volhard C (1998). fork head domain genes in zebrafish. Dev Genes Evol.

[B76] Krauss S, Concordet JP, Ingham PW (1993). A functionally conserved homolog of the Drosophila segment polarity gene hh is expressed in tissues with polarizing activity in zebrafish embryos. Cell.

[B77] Appel B, Korzh V, Glasgow E, Thor S, Edlund T, Dawid IB, Eisen JS (1995). Motoneuron fate specification revealed by patterned LIM homeobox gene expression in embryonic zebrafish. Development.

[B78] Krauss S, Johansen T, Korzh V, Fjose A (1991). Expression pattern of zebrafish pax genes suggests a role in early brain regionalization. Nature.

[B79] Neyt C, Jagla K, Thisse C, Thisse B, Haines L, Currie PD (2000). Evolutionary origins of vertebrate appendicular muscle. Nature.

[B80] Schulte-Merker S (2002). Looking at embryos. Zebrafish: a practical approach.

[B81] Davis GK, D'Alessio JA, Patel NH (2005). Pax3/7 genes reveal conservation and divergence in the arthropod segmentation hierarchy. Dev Biol.

[B82] Hoffman L, Miles J, Avaron F, Laforest L, Akimenko MA (2002). Exogenous retinoic acid induces a stage-specific, transient and progressive extension of Sonic hedgehog expression across the pectoral fin bud of zebrafish. Int J Dev Biol.

