# Supplementary figures and images for: Genetic analysis of the two zebrafish patched homologues identifies novel roles for the hedgehog signaling pathway
Source: BMC Dev Biol. 2008 Feb 19;8:15. doi: 10.1186/1471-213X-8-15 (PMC2275722; doi:10.1186/1471-213X-8-15)

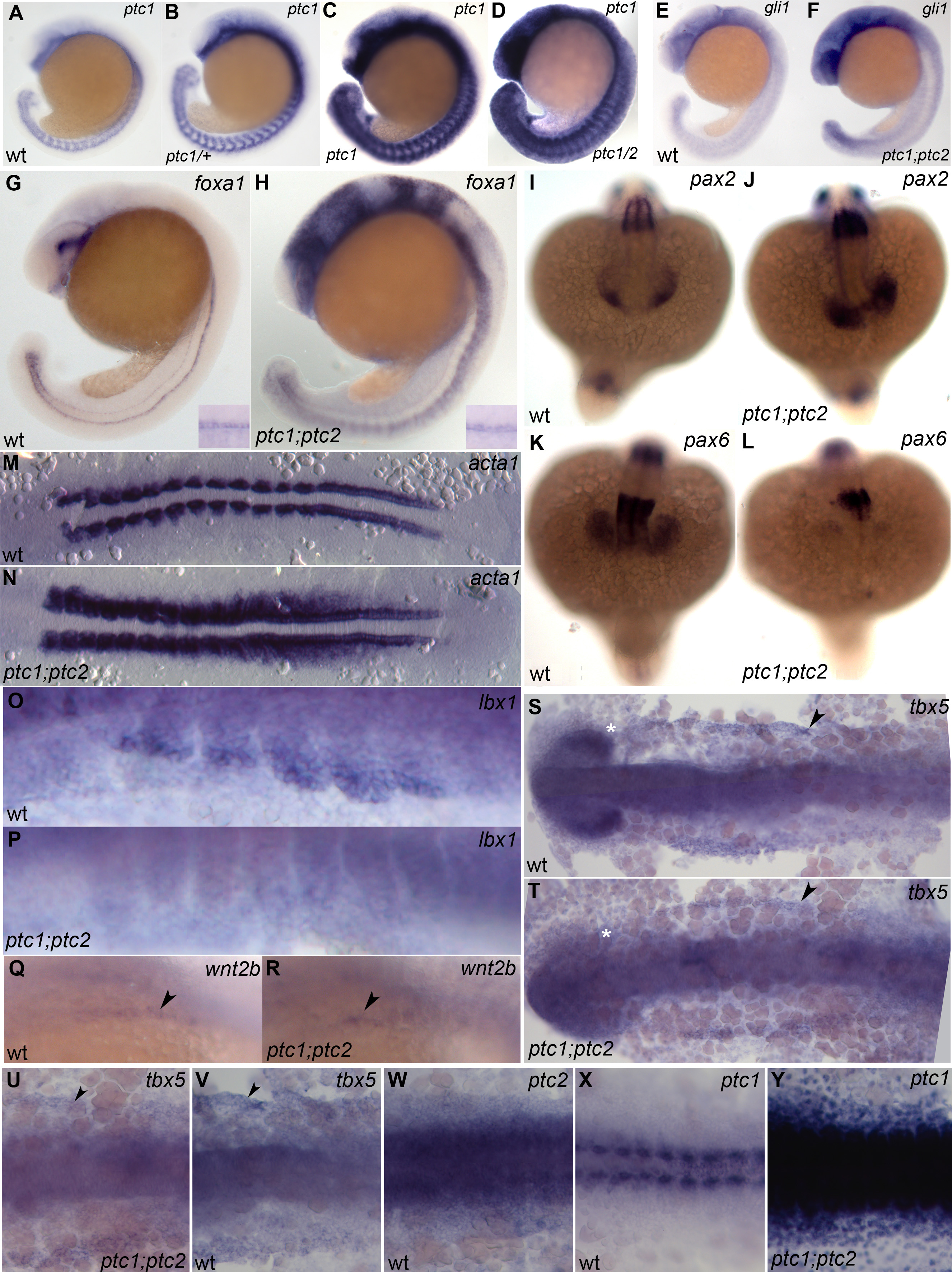

Supplement: Additional file 1 — A,B,C,D ptc1 expression in wild-type (A), ptc1/+ (B), ptc1 mutant (C) and ptc1;ptc2 mutant (D) stained in a single reaction 19 somite stage; no significant increase is detectable in ptc2 mutants at this stage. E,G,I,K,M,O,Q,S,V,W,X) wild type embryos; F,H,J,L,N,P,R,T,U,Y) ptc1;ptc2 mutants. E,F) 21 somite stage; Gli expression is increased ptc1;ptc2 mutants. G,H) foxa1 expression labels medial and lateral floor plate and is expanded in ptc1;ptc2 mutants, shh a medial floor plate marker, remains unchanged (inset). I,J,K,L) 18 somite stage; pax2 labeling of the optic stalk in wild type (I) is expanded in ptc1;ptc2 mutants (J). At the same time pax6 expression in the optic cup is severly reduced in ptc1;ptc2 mutants (K,L). M,N) 12 somite stage, flat mount, dorsal view; skeletal muscle alpha actin1, a marker for muscle differentiation is increased in ptc1;ptc2 mutants. O,P) 20 somite stage, ventral part of anterior somites. Presumptive migratory myoblasts that will form fin muscle express lbx1. Expression of this gene is lost in ptc1;ptc2 mutants. Q,R) Oblique view on wnt2b expression focusing on region between lateral plate and ventral somites 21 somite stage. This gene is required for fin bud formation and is still expressed in ptc1;ptc2 mutants. S,T) 10 somite stage, flat mount, dorsal view. Initial expression of tbx5 in the -then continuous- heart and pectoral fin primordium is unaltered in ptc1;ptc2 mutants (arrowheads). Double mutants lack tbx5 expression in the optic cup (star). U,V,W,X,Y) 10 somite stage flat mount dorsal view U,V) ptc1;ptc2 mutants and wild-type show tbx5 labeling at the edge of the lateral plate mesoderm, ptc2 is also expressed in this region (W). ptc1 expression is normally only detectable in the somites strongly in the adaxial cells and weakly in the lateral somite. However, ptc1 is upregulated throughout the entire embryo in ptc1;ptc2 mutants, showing that it can respond to Hh signaling in lateral plate mesoderm. Note: X and Y were stained [file 1471-213X-8-15-S1.jpeg]

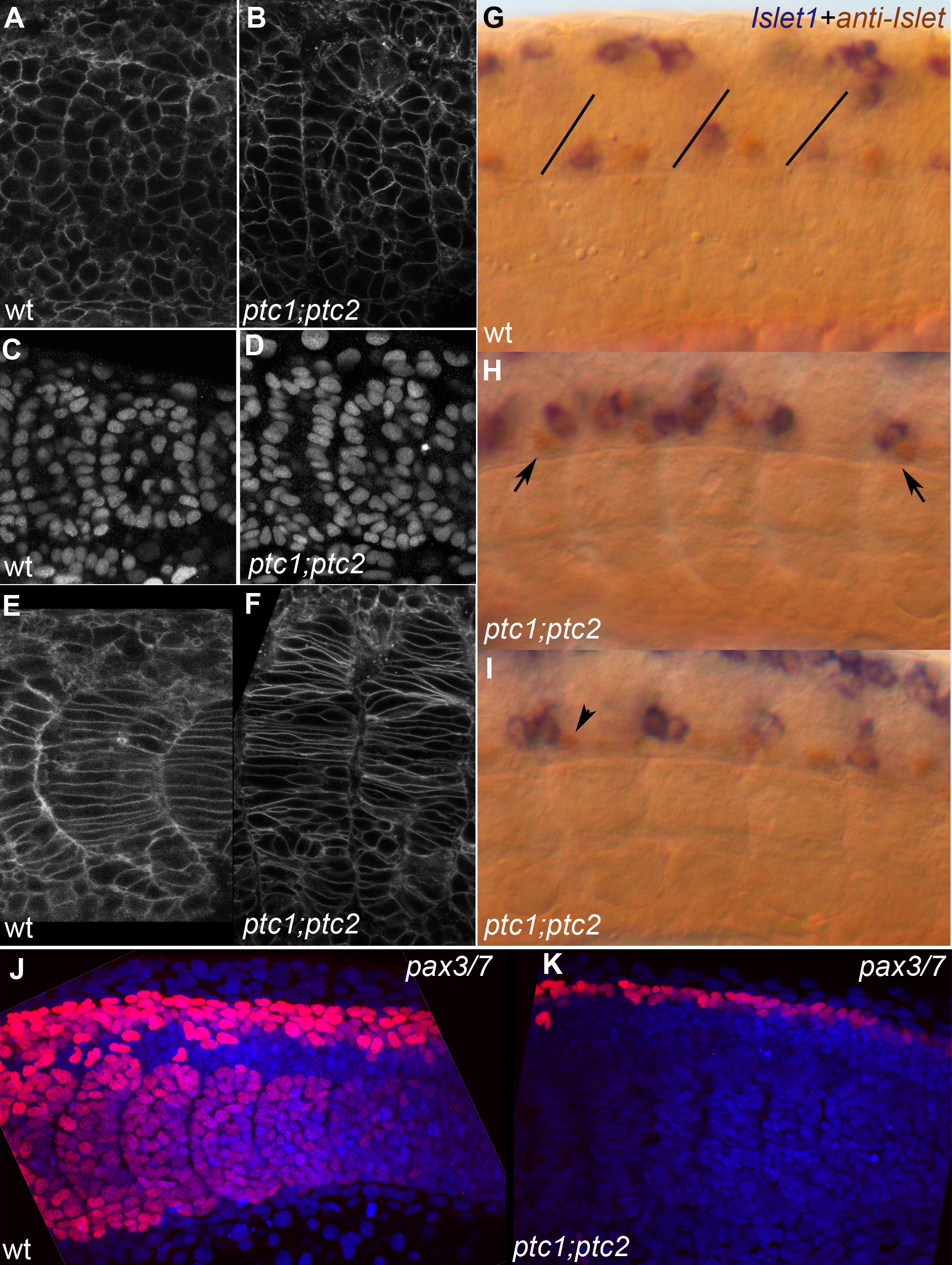

Supplement: Additional file 2 — A,C,E,G,J) wild type embryo; B,D,F,H,I,K) ptc1;ptc2 mutants. A,B) lynGFP membrane label showing most recent fully formed somite, 14 somite stage. Double mutant somites still form an epithelium but irregularities in the epithelial somite are more frequent. C,D) DAPI nuclear stain showing essentially the same result as A,B. E,F) LynGFP labeling outlining cells of differentiating somites in a 14 somite wild-type and ptc1;ptc2 mutant embryo, medial optical section through somite 10 and 9+10, respectively. Somites have lost their clear V shape and the number of elongated adaxial cells appears increased. G,H,I) Double labeling showing anti-Islet (brown) and islet1 expression (20 ss). Brown cells express only Islet 2 and are Caudal Primary (CaP) neurons, blue/brown cells express islet 1 (and possibly 2) and are Middle Primary neurons (MiP). In wild-type (G) brown CaPs are located in the middle of each segment whereas the blue/brown MiPs are close to the somite boundary (drawn-in in G for clarity) [54]. In a ptc1;ptc2 mutant background (H,I) mistakes in this order are very frequent, for instance, blue and brown cells close to the posterior of the segment, or the exact mirror image of that (H, arrows). In (I) a brown cell can be seen in the position where a blue cell would be expected (arrowhead). J,K) Z-projection of Pax3/7 labeling (red) and nuclear DAPI stain (blue) on posterior 5–6 somites of 20 somite stage embryos showing the loss of all pax3/7 staining in the somites, and a reduction of the number of pax3/7 positive nuclei in the dorsal neural tube in ptc1;ptc2 double mutants. [file 1471-213X-8-15-S2.jpeg]
